# The Mediating Roles of Two Psychological Experience Pathways: How Cross-Border Live Streaming Interactions Trigger Impulsive Purchases?—The Moderating Effect of AI Enablement

**DOI:** 10.3390/bs16091693

**Published:** 2026-09-20

**Authors:** Yangxue Xiang, Miaoli Liu, Jin Chen, Zhaoxu Chen

**Affiliations:** 1 Alibaba School of Business, Hangzhou Normal University, Hangzhou 311121, China; 2 Research Center for Technological Innovation, Tsinghua University, Beijing 100084, China

**Keywords:** cross-border e-commerce, live streaming interaction, flow experience, AI enablement, impulsive purchase intention, SOR theory

## Abstract

With the booming of cross-border live streaming e-commerce, this study examined the influence mechanism of cross-border live streaming interactions on consumers’ impulsive purchase intention based on the timulus–organism–response (SOR) model. Two independent psychological organism mediators are proposed, immersive experience and foreign cultural identity, and AI (artificial intelligence) enablement acts as a cross-layer moderating variable. Three core dimensions of cross-border live streaming interaction (consumer–product, consumer–consumer, consumer–anchor) are refined, and empirical tests are conducted with 620 valid questionnaires via SPSS 26.0, AMOS 28.0 and PROCESS. Results showed that consumer–product and consumer–anchor interactions exerted significant positive direct effects on impulsive purchase intention (consumer–consumer interaction’s direct effect was insignificant); both flow experience dimensions exert partial mediating effects in the paths linking consumer–product/consumer–anchor interaction to impulsive purchase intention and full mediating effects for the consumer–consumer interaction path. AI enablement positively moderates the effect of live streaming interactions on flow experience and the effect of immersive experience on impulsive purchase intention, but its moderating effect on the foreign cultural identity–impulsive purchase intention link was insignificant. This study advanced the research on cross-border live streaming consumer behavior and offered targeted practical suggestions for platform optimization with AI technology while enriching the SOR model’s application in cross-cultural consumption scenarios.

## 1. Introduction

Recent data released by the Ministry of Commerce of China shows that the total import and export volume of China’s cross-border e-commerce reached 2.38 trillion yuan in 2023, a year-on-year increase of 15.6%. Meanwhile, the market scale of China’s cross-border live-streaming e-commerce hit 284.58 billion yuan in 2023 and is projected to reach 828.70 billion yuan by 2025 with its rapid development. Real-time interactivity constituted a core competitive edge of live commerce in prior research (L. Wang et al., 2024). Scholars demonstrated that such interactivity boosted consumer engagement, heightened perceived timeliness and delivered personalized shopping experiences, which further exerted prominent effects on viewers’ impulsive purchase intention (J. Chen et al., 2024). Furthermore, prior evidence indicated that live streaming triggered consumers’ flow states (Y. P. Jiang et al., 2024). This psychological experience fostered emotional bonding and cultural identification, alongside the arousal of positive affective responses, which collectively shortened the cognitive pathway toward impulsive buying (Dong et al., 2023; Huang et al., 2023; M. Wang et al., 2021).

However, interactive mechanisms within cross-border live commerce differ drastically from domestic live broadcast formats. Spatial and temporal separation may lead to information opacity in cross-border online shopping, which in turn affects consumer decision-making (Zhou et al., 2018). In addition, existing studies on cross-border e-commerce live streaming marketing strategies and consumer purchase intention mainly take anchor characteristics (Wen et al., 2024), perceived value (Xu et al., 2023) and perceived trust (Ling et al., 2024) as mediating variables based on the SOR theory. Although it has been proven that consumers exhibit higher attention to live streaming in a state of flow (C. C. Chen & Lin, 2018), empirical research on the impact of cross-border e-commerce live streaming interactions on consumers’ flow experience remains insufficient (Y. P. Jiang et al., 2024; J. Chen et al., 2024).

Meanwhile, the adoption of artificial intelligence represented a pivotal developmental trend within live-streaming e-commerce. Prior researchers leveraged AI algorithms to resolve real-time machine translation barriers and deployed the hybrid framework of human anchors paired with virtual IP assistants to enrich live broadcast attractiveness (J. Y. Gao et al., 2024; N. Zhang, 2023). Despite the growing prevalence of AI applications in live commerce, prior empirical studies remain insufficient to unpack whether AI tools exert moderating effects on the linkage between cross-border live streaming interactions and consumers’ impulsive purchase intention.

This study intended to answer three core research questions as follows. First, what specific modes of cross-border e-commerce live streaming interactions can effectively stimulate consumers’ flow experience? Second, how does flow experience influence consumers’ impulsive purchase intention in the context of cross-border e-commerce live streaming interactions? Finally, how do AI technologies moderate the causal chain of “cross-border live streaming interaction → flow experience → impulsive purchase intention” constructed under the SOR theoretical framework?

Based on a comprehensive literature review, this study distinguishes two core psychological states generated by cross-border live streaming viewing, namely immersive experience and foreign cultural identity, and treats them as two separate mediating variables in the SOR causal chain. We further extract three core dimensions of cross-border e-commerce live streaming interaction and conduct empirical analysis via SPSS 26.0, AMOS 28.0 and PROCESS. Structural equation modeling is employed to test direct, mediating and moderating effects, thereby revealing the intrinsic relationships among the variables. This study advanced the comprehension of consumer decision-making mechanisms within cross-border live commerce and delivered actionable insights for practitioners to design targeted operational tactics that capture audience attention and lift sales performance.

## 2. Theoretical Framework and Hypotheses

### 2.1. Cross-Border E-Commerce Live Streaming Interaction

Prior literature defined live streaming interactivity as a set of real-time communication functions integrated via text, audio and video channels to deliver authentic and reliable information to consumers, helping them accurately evaluate product quality (Zhou et al., 2018; De Wit et al., 2020). Consumers’ perceived interactivity from real-time consumer–anchor interaction during live streaming viewing can enhance their purchase intention (W. G. Sun et al., 2021).

Scholars have investigated interaction modes from both functional and perceptual perspectives. From a functional perspective, Liberman et al. (2007) categorized interaction into human–computer interaction (focusing on human–media interaction) and interpersonal interaction (focusing on human–human interaction), and Nambisan and Baron (2009) further added the dimension of consumer–product interaction. From a perceptual perspective, Kang et al. (2020) explored dynamic interaction behaviors in live e-commerce by taking responsiveness and personalization as two dimensions of perceived interactivity, which were found to affect the strength of social connection and user engagement. C. Q. Jiang et al. (2019) divided live streaming interactions into consumer–consumer, consumer–merchant and consumer–product interactions to examine their effects on consumer trust and shopping intention. Kang et al. (2020) explained the formation mechanism of customer purchase intention in social e-commerce from the perspectives of consumer–product interaction and interpersonal interaction. F. Liu et al. (2022) classified live streaming interactions into three dimensions, information–obtaining interaction, purchase dynamics–tracking interaction and monetary incentive–obtaining interaction, and verified the promotional effect of these interactions on consumers’ purchase intention. Luo et al. (2024) explored the impact of customer interaction information on impulsive purchase in live e-commerce streaming from three aspects: product information quality, live streaming interaction quality and live streaming credibility. Ling et al. (2024) used the SOR framework to reveal the effects of interpersonal interactions (consumer–anchor and consumer–consumer interactions) on psychological distance and consumer purchase intention.

Drawing on the classification of interaction modes by C. Q. Jiang et al. (2019), Kang et al. (2020) and Ling et al. (2024), this study generalizes cross-border e-commerce live streaming interaction into three core dimensions: consumer–product interaction, consumer–consumer interaction and consumer–anchor interaction.

### 2.2. AI Enablement

Artificial intelligence has driven the further development of live streaming e-commerce (N. Zhang, 2023). Live streaming platforms can leverage AI technologies to improve users’ interactive experience and enhance their social engagement (Wen et al., 2024). AI-enabled e-commerce platforms can provide personalized product and live streaming content recommendations based on consumers’ browsing and shopping records through data processing and intelligent recommendation algorithms, thereby boosting user engagement (Paschen et al., 2019).

Meanwhile, AI technology plays a vital role in optimizing the virtual reality experience, allowing users to experience virtual product displays and shopping environments and thus enhancing the immersion and attractiveness of live streaming interactions (Kautish & Khare, 2022). AI chatbots possess powerful search and comparison capabilities in live e-commerce streaming (Zhu et al., 2022); through intelligent speech recognition and machine learning algorithms, AI can automatically answer viewers’ common questions, reducing the workload of human customer service (Baabdullah et al., 2022). In addition, AI can be applied to virtual anchors in live streaming to cut human resource costs (J. Y. Gao et al., 2024). AI technology breaks through temporal and spatial barriers (Y. X. Zhang et al., 2023), assisting anchors in intelligently recommending products and answering viewers’ questions in real time. It also provides virtual image and role-playing functions (Khare et al., 2023), bringing a new interactive experience to stimulate audience interest and engagement. Intelligent robots can interact with viewers through facial recognition, voice recognition and other technologies (Zhu et al., 2022), providing an unbiased and high-quality service experience that enhances viewer satisfaction and engagement.

### 2.3. Flow Experience

Within internet research contexts, scholars examined flow experience from unidimensional and multidimensional lenses. From the unidimensional viewpoint, Hoffman and Novak (1996) and H. Chen et al. (1999) explored users’ flow states within online environments. Luna et al. (2002) constructed a theoretical model to capture consumers’ flow experience on cross-cultural webpages. Oh et al. (2012) adopted Kano’s model to analyze the attributes of e-shopping platforms and identified that flow experience strengthened consumers’ platform trust. Hyun et al. (2022) further validated flow experience’s predictive effect on consumers’ shopping behaviors across social media platforms.

From the multidimensional perspective, Skadberg and Kimmel (2004) split flow experience into temporal dissociation and hedonic pleasure to evaluate visitors’ psychological states during website browsing. Z. Jiang and Benbasat (2004) classified flow experience into three dimensions—perceived control, immersion, and shopping enjoyment—and tested how visual and functional product controls shaped shoppers’ flow perceptions amid e-commerce scenarios. Following the rise in mobile internet technology, scholars incorporated flow experience into live-streaming consumer behavior research (W. G. Sun et al., 2021; Dong et al., 2023). Prior empirical work confirmed that consumers devoted greater attention to live broadcasts once immersed in flow, and such immersive states fostered emotional bonding and cultural recognition (C. C. Chen & Lin, 2018; Dong et al., 2023).

Classical flow theories focus on situational absorption, temporal distortion and self-transcendence in single-culture online scenarios. However, these theories ignore unique cross-cultural emotional connections generated when consumers watch cross-border live streams. For cross-border shoppers, live broadcast stimuli simultaneously activated two distinct psychological perceptions: immersive experience and foreign cultural identity. Foreign cultural identity represented long-run cross-cultural affection instead of core flow-state traits such as absorption and temporal dissociation. Accordingly, this study refrained from aggregating the two constructs into a higher-order flow factor and instead treated immersive experience and foreign cultural identity as two separate organism mediators embedded within the SOR framework.

### 2.4. SOR Model Theory

The SOR model, rooted in environmental psychology, was first proposed by Mehrabian and Russell (1974). Derived from the behaviorist stimulus–response (S-R) model, the SOR model consists of three components: stimulus, organism and response. Eroglu et al. (2001) applied the SOR model to explore the impact of online store atmosphere on consumers’ personal feelings and perceptions, arguing that the stimulus dimension in online store atmosphere is reflected in all external perceptions of consumers, shopping tasks or emotional reactions triggered by consumers’ social relationships. The organism dimension refers to a series of cognitive and emotional processing processes of consumers after receiving stimuli, and the response dimension refers to consumers’ approach or avoidance behaviors, including impulsive purchase intention and actual purchase behavior. This is the first time that scholars have applied the SOR model to the online retail context.

Since then, the SOR model has been widely used in the field of online consumer behavior research, including studies on consumers’ impulsive purchase intention, continuous use intention and information adoption behavior (Hyun et al., 2022; Molinillo et al., 2021; W. G. Sun et al., 2021). X. X. Wang et al. (2024) distinguished between environmental stimuli and consumer behavior and constructed a conceptual model and research hypotheses based on the SOR framework to explore the relationships among usability, information validity, emotional arousal and impulsive purchase intention. B. B. Sun et al. (2023) combined the SOR model and Schachter–Singer (SST) theory to empirically study the relationship between time pressure and impulsive purchase from both emotional and cognitive perspectives. H. L. Gao et al. (2022) built a live streaming impulsive purchase model based on the SOR theory and explored the effects of ambient cues and promotional cues on online consumers’ impulsive purchase intention through the mediating roles of emotion and the middle-of-the-road tendency. Li et al. (2024) used the SOR framework to construct a model where anchors’ personal charisma, professionalism, interactivity and entertainment influence consumers’ impulsive purchase intention by shaping their trust and flow experience.

### 2.5. Impulsive Purchase Intention

Impulsive purchase intention is defined as a strong and sudden desire to purchase a product that arises when a consumer watches a live streaming session (Lu & Chen, 2021). Negash et al. (2024) found that rewards, recognition, evaluations and ratings are the key drivers of perceived value, which has a significant positive correlation with impulsive behavior. Safeer (2024) demonstrated that social media marketing campaigns exert a significant impact on brand resonance, consumer emotional responses and online impulsive purchase intention.

In live streaming commerce, impulsive purchases are triggered by unique environmental cues, such as limited live streaming time, real-time interaction with persuasive anchors, and comprehensive product demonstrations. These cues enhance consumers’ sense of urgency and establish an emotional connection with them (Lo et al., 2022). Ming et al. (2021) examined the factors influencing consumers’ impulsive purchase from the perspectives of live streaming viewers and suppliers. L. Chen et al. (2024) proposed a live streaming sales channel selection strategy based on consumers’ impulsive purchase characteristics. Ma et al. (2023) explored the effects of platform attributes, interaction strategies and parasocial relationships on impulsive purchase in live streaming commerce. Given the complexity and difficulty of measuring actual impulsive purchase behavior (Chan et al., 2016), most studies use impulsive purchase intention as a proxy variable for actual purchase behavior (Parboteeah et al., 2009; Y. Liu et al., 2013).

### 2.6. Research Hypotheses and Theoretical Model

#### 2.6.1. Cross-Border E-Commerce Live Streaming Interaction and Impulsive Purchase Intention

Rich product information and instant interactive feedback can improve the accuracy of consumers’ impulsive purchase decision-making and enhance brand loyalty (Y. P. Jiang et al., 2024; Mou et al., 2020). Such interactions go beyond simple information transmission and integrate multiple dimensions, including product display, information exchange, emotional resonance and social elements, aiming to improve consumers’ shopping experience and stimulate their impulsive purchase intention (Wen et al., 2024). This study selects consumer–product interaction, consumer–consumer interaction and consumer–anchor interaction as the key variables to measure cross-border e-commerce live streaming interaction.

Consumer–product interaction refers to the all-round and multi-angle presentation of product value in cross-border e-commerce live streaming rooms, which helps consumers better understand product characteristics and thus form more satisfactory impulsive purchase intentions; when consumers perceive sufficient product value, they are more likely to generate purchase intention (F. Liu et al., 2022). Consumer–consumer interaction provides valuable user-generated content (Chang, 2023), and the group effect and social identity mechanism formed in live streaming further promote the formation of impulsive purchase intention. Through their professionalism, affinity and communication skills, anchors reduce the perceived uncertainty of cross-border shopping (Guo et al., 2021), establish trust with viewers, and enhance their shopping experience and satisfaction.

Accordingly, the following hypotheses are proposed:

**H1a.**  *Consumer–product interaction in cross-border e-commerce live streaming has a positive effect on impulsive purchase intention*.

**H1b.**  *Consumer–consumer interaction in cross-border e-commerce live streaming has a positive effect on impulsive purchase intention*.

**H1c.**  *Consumer–anchor interaction in cross-border e-commerce live streaming has a positive effect on impulsive purchase intention*.

#### 2.6.2. Cross-Border E-Commerce Live Streaming Interaction and Flow Experience

Previous research has found that the real-time interactivity and immersion of live streaming make it more attractive to consumers than traditional online shopping models (Ling et al., 2024). As a major form of synchronous social media, cross-border e-commerce live streaming features high real-time performance, strong interactivity and synchronous communication (Zhou et al., 2018), which can improve the efficiency of sales services. In addition, the visibility of cross-border e-commerce live streaming can enhance information transparency (Xu et al., 2022) and reduce consumers’ perceived risk and uncertainty. The real-time response and interaction in cross-border e-commerce live streaming create a lively shopping atmosphere, which attracts consumers to purchase cross-border products by focusing their perception and attention on live streaming content and inducing a flow experience (Y. Sun et al., 2019).

The country-of-origin image of products affects consumers’ value judgment, while product brands, stories and cultural connotations influence their emotional identification (Bao et al., 2022). Consumer–consumer interaction increases the fun and engagement of shopping through sharing shopping experiences, asking questions and seeking suggestions; it also helps build trust among consumers by providing product information, recommendations and evaluations (F. Liu et al., 2022). Anchors are not only product promoters but also affable and influential image spokespersons, whose image affects consumers’ trust in products (Terasaki et al., 2022). Their interactive behaviors can shape consumers’ psychological states: interactive communication with anchors not only provides professional product information and purchase advice but also establishes emotional connections and attachment with consumers through emotional communication and the introduction of cultural backgrounds (Kang et al., 2020), narrows the psychological distance between consumers and anchors (Luo et al., 2024), and thus improves consumers’ flow experience in live shopping.

Accordingly, the following hypotheses are proposed:

**H2a.**  *Consumer–product interaction in cross-border e-commerce live streaming has a positive effect on immersive experience*.

**H2b.**  *Consumer–consumer interaction in cross-border e-commerce live streaming has a positive effect on immersive experience*.

**H2c.**  *Consumer–anchor interaction in cross-border e-commerce live streaming has a positive effect on immersive experience.*

**H2d.**  *Consumer–product interaction in cross-border e-commerce live streaming has a positive effect on foreign cultural identity.*

**H2e.**  *Consumer–consumer interaction in cross-border e-commerce live streaming has a positive effect on foreign cultural identity.*

**H2f.**  *Consumer–anchor interaction in cross-border e-commerce live streaming has a positive effect on foreign cultural identity.*

#### 2.6.3. The Mediating Role of Flow Experience

It has been proven that consumers pay more attention to live streaming in a state of flow (C. C. Chen & Lin, 2018) and are more likely to establish emotional connections and identification (Dong et al., 2023). The impact of flow experience on impulsive purchase intention in cross-border e-commerce live streaming is reflected in two key dimensions. First, immersive experience enables consumers to deeply participate in live streaming content by enhancing their understanding of products and brands and creating an immersive shopping experience (Liao et al., 2023), making them more likely to generate purchase intention. Consumers generate positive emotions through interaction and information acquisition in live streaming (Huang et al., 2023), which further stimulates impulsive purchase intention. In cross-border e-commerce live streaming, interactions help consumers comprehensively understand product features, shortening the psychological process of forming impulsive purchase intention (M. Wang et al., 2021).

Second, foreign cultural identity refers to a psychological state in which consumers emotionally identify with the culture of another country. Sympathy and attachment to a specific country affect consumers’ trust and evaluation of its products, and irrational factors such as emotional identification with or hostility toward a foreign country have a significant impact on their impulsive purchase intention (Bozdag & Durmus, 2024). In cross-border e-commerce live streaming, the display of exotic cultural elements, brand stories and cultural connotations enhances the emotional connection between consumers and products; the exchange of product and cultural stories between anchors and consumers helps consumers establish identification with a specific culture or country (Bozdag & Durmus, 2024).

Accordingly, the following hypotheses are proposed:

**H3a.**  *Immersive experience mediates the positive link between consumer–product interaction and impulsive purchase intention in cross-border live streaming e-commerce; foreign cultural identity plays another independent mediating role in the same path.*

**H3b.**  *Immersive experience mediates the positive link between consumer–consumer interaction and impulsive purchase intention in cross-border live streaming e-commerce; foreign cultural identity plays another independent mediating role in the same path.*

**H3c.**  *Immersive experience mediates the positive link between consumer–anchor interaction and impulsive purchase intention in cross-border live streaming e-commerce; foreign cultural identity plays another independent mediating role in the same path.*

**H3d.**  *Foreign cultural identity mediates the positive link between consumer–product interaction and impulsive purchase intention in cross-border live streaming e-commerce; immersive experience plays another independent mediating role in the same path.*

**H3e.**  *Foreign cultural identity mediates the positive link between consumer–consumer interaction and impulsive purchase intention in cross-border live streaming e-commerce; immersive experience plays another independent mediating role in the same path*.

**H3f.**  *Foreign cultural identity mediates the positive link between consumer–anchor interaction and impulsive purchase intention in cross-border live streaming e-commerce; immersive experience plays another independent mediating role in the same path.*

#### 2.6.4. The Moderating Role of AI Enablement

In cross-border e-commerce live streaming, AI technology plays a crucial role in moderating and enhancing the effects of live streaming interactions on consumers’ flow experience and impulsive purchase intention (Sidlauskiene et al., 2023; Kautish & Khare, 2022; Baabdullah et al., 2022).

There are essential differences in the formation logic of immersive experience and foreign cultural identity. Immersive experience represented transient situational feelings triggered by real-time live broadcast ambiance, which could be effectively enhanced via AI real-time auxiliary functions including multilingual interpretation and virtual product visualization. In contrast, foreign cultural identity reflected stable long-run emotional attachment shaped by prolonged exposure to cross-cultural content. Short-term AI live-stream tools merely improved instant interactive scenarios and failed to reshape consumers’ deep-rooted cross-cultural perceptions. Accordingly, this study proposed the exploratory hypothesis H4d and anticipated that AI exerted no statistically significant moderating influence on the foreign cultural identity–impulsive purchase intention pathway.

Accordingly, the following hypotheses are proposed:

**H4a.**  *AI enablement positively moderates the respective effects of consumer–product interaction, consumer–consumer interaction, and consumer–anchor interaction on immersive experience.*

**H4b.**  *AI enablement positively moderates the respective effects of consumer–product interaction, consumer–consumer interaction, and consumer–anchor interaction on foreign cultural identity.*

**H4c.**  *AI enablement positively moderates the link between immersive experience and impulsive purchase intention.*

**H4d.**  *AI enablement positively moderates the link between foreign cultural identity and impulsive purchase intention.*

#### 2.6.5. Theoretical Model

**Figure 1:**
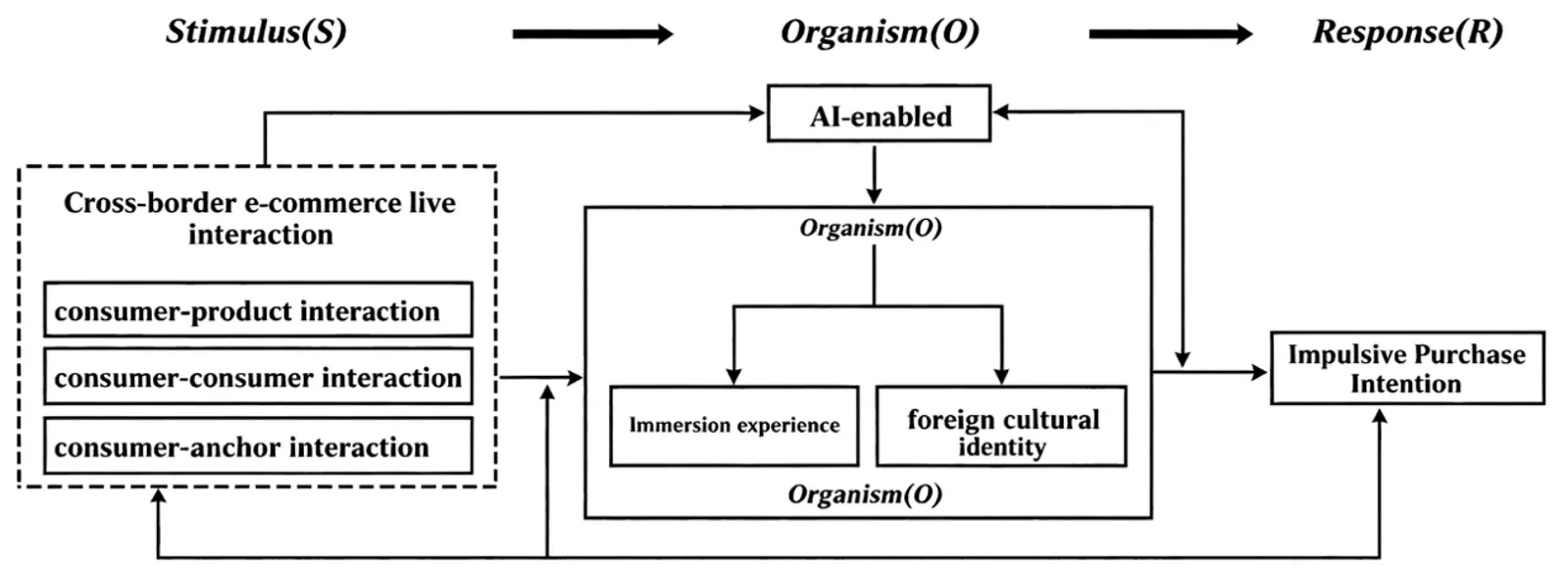
Theoretical model: cross-border live streaming interactions, dual independent mediators and AI moderator.

Based on the above research hypotheses, this study constructs a theoretical model of the influence of cross-border e-commerce live streaming interaction on impulsive purchase intention, with flow experience as the mediator and AI enablement as the moderator (Figure 1).

- Stimulus (S): Cross-border e-commerce live streaming interaction (consumer–product interaction, consumer–consumer interaction, consumer–anchor interaction).
- Organism (O): Flow experience (immersive experience, foreign cultural identity); AI enablement (moderator).
- Response (R): Impulsive purchase intention.

## 3. Methods

### 3.1. Measurement Scale Development

This research utilized questionnaire-based data collection to collect data from individuals who have experience with cross-border e-commerce live streaming shopping. Respondents were asked to recall their most recent cross-border e-commerce live streaming shopping experience and answer the questionnaire based on their actual perceptions and experiences. All measurement items were adapted from mature scales in existing literature and adjusted appropriately to fit the research context of cross-border e-commerce live streaming. A 7-point Likert scale was used for all items (1 = strongly disagree, 7 = strongly agree).

### 3.2. Data Collection

As China is one of the world’s largest cross-border e-commerce markets with a huge online consumer base and a mature cross-border e-commerce ecosystem, this study focuses on Chinese consumers of cross-border e-commerce live streaming. The online questionnaire was designed using Wenjuanxing (https://www.wjx.cn/), a widely used data collection platform in China. We distributed questionnaires across multiple online platforms from January to July 2024, including Weibo, WeChat, Douban, Taobao groups and TikTok, and collected a total of 1279 responses.

Detailed invalid sample elimination criteria are defined as follows: (1) questionnaires with all items filled with identical scores; (2) completion time shorter than 90 s, indicating careless random filling; and (3) missing answers over 20% of total measurement items. A total of 659 questionnaires were eliminated based on the above rules, leaving 620 valid questionnaires, with an effective response rate of 48.5%. The high invalid rate mainly originates from casual perfunctory filling of questionnaire traffic samples from short-video platform promotion.

### 3.3. Data Analysis Methods

We deployed SPSS 26.0 to conduct descriptive statistics and reliability examinations. We supplemented reliability evaluation with Cronbach’s α and McDonald’s Omega coefficients. We then ran AMOS 28.0 to carry out confirmatory factor analysis (CFA). This step verified the scale’s construct, convergent and discriminant validity. We also applied structural equation modeling (SEM) to examine direct paths among latent variables. For moderated mediation analyses, we utilized the SPSS PROCESS macro. Following Hayes’ theoretical framework, we uniformly adopted Model 58 instead of separate Model 4 and Model 5. We set 5000 bootstrap iterations and a 95% confidence interval (CI) while treating gender, age, education, occupation and monthly disposable income as control variables.

Prior to generating interaction terms for moderation tests, we grand-mean-centered all predictor and moderator variables. This procedure mitigated multicollinearity derived from product-term calculations.

We measured all survey items on a 7-point ordered Likert scale. From a theoretical perspective, polychoric correlation paired with diagonal weighted least squares (DWLS) represents the ideal estimation method for ordinal categorical data. Nevertheless, mainstream software including AMOS 28.0, SPSS 26.0 and the PROCESS 5.0 macro lacks native support for DWLS based on polychoric correlations. For this reason, we chose maximum likelihood estimation as our primary analytical approach. We elaborated on this methodological limitation within the study’s limitation section.

### 3.4. Ethics Statement

All study procedures complied with the ethical standards of the institutional research committee and the 1964 Helsinki Declaration and its subsequent amendments.

We administered a completely anonymous online survey and collected no identifying or sensitive personal information from respondents. An information sheet describing the study purpose, procedures, risks, benefits, and confidentiality measures was presented on the first page of the survey; participants were notified that participation was voluntary and that they could discontinue their involvement at any time without adverse consequences, and implied consent was obtained by survey completion.

Per the Measures for Ethical Review of Life Sciences and Medical Research Involving Human Subjects (2023) released by China’s National Health Commission (National Health Commission of China et al., 2023), studies relying on anonymized, non-harmful data without sensitive personal information or commercial interests qualify for ethical-review exemption. Accordingly, the Institutional Review Board of Hangzhou Normal University granted a formal ethical-approval waiver for the present study, and relevant exemption documentation was kept for institutional filing.

## 4. Results

### 4.1. Descriptive Statistics

The results are shown in Table 1.The 620 valid respondents were mainly female (58.4%), were aged 18–30 years (77.1%), had a bachelor’s/associate degree (62.3%), and were employed in enterprises and institutions (56.8%). In terms of cross-border live streaming shopping behavior, 41.1% of respondents watched 1–3 times per week, 46.9% purchased 1–2 times per month, and 27.9% spent 101–500 yuan per month on average. Structural equation modeling requires the sample size to be more than 10 times the number of measurement items (Xiao et al., 2019; Kline, 2011), and the 620 samples in this study were sufficient to meet the statistical requirements of the research.

### 4.2. Reliability and Validity Analysis

#### 4.2.1. Reliability Analysis

The results are shown in Table 2. SPSS 26.0 was used to test the internal consistency reliability of the scale, with Cronbach’s α coefficient as the evaluation index; McDonald’s Omega coefficients were supplemented for robust reliability verification per peer review suggestion. The results showed that the Cronbach’s α coefficients of all variables (consumer–product interaction: 0.889; consumer–consumer interaction: 0.861; consumer–anchor interaction: 0.874; AI enablement: 0.805; immersive experience: 0.838; foreign cultural identity: 0.840; impulsive purchase intention: 0.858) were all greater than the critical value of 0.7, and the overall Cronbach’s α coefficient of the scale is 0.910, indicating that the scale had good internal consistency and reliability.

#### 4.2.2. Validity Analysis

AMOS 28.0 was used to conduct confirmatory factor analysis (CFA) on the 28 measurement items to test the construct validity, convergent validity and discriminant validity of the scale.

The results are shown in Table 3. Construct Validity: The chi-square/degree of freedom (χ^2^/df) was 1.469 (less than 3); GFI (0.955), AGFI (0.943), NFI (0.956), IFI (0.986), TLI (0.983) and CFI (0.985) are all greater than 0.9, and RMSEA (0.028) and RMR (0.034) were both less than 0.05. Partial model paths were adjusted according to modification indices to optimize fitting indicators; the detailed modified paths are listed in Appendix A. All fit indices met the recommended standards, indicating good construct validity.

The results are shown in Table 4. Convergent Validity: Evaluated by composite reliability (CR) and average variance extracted (AVE), the CR values of all variables were greater than 0.7, and the AVE values are all greater than 0.5, indicating good convergent validity.

The results are shown in Table 5.Discriminant Validity: Tested by comparing the square root of the AVE of each variable with the correlation coefficients between variables, the square root of the AVE of each variable was greater than the absolute value of the correlation coefficient between the variable and other variables, indicating good discriminant validity.

#### 4.2.3. Common Method Bias Test

To eliminate common method variance bias from the single-source self-reported questionnaire, two classic detection approaches were adopted.

First was Harman’s single-factor test. All 28 measurement items were put into unrotated exploratory factor analysis; the maximum single extracted factor explained 23.71% of the total variance, below the critical threshold of 40%, effectively ruling out serious common method bias threats.

Second was the latent marker variable technique, which selected four irrelevant filler items as marker variable and embedded the latent marker into the full measurement model; the change in model path coefficients before and after adding the marker was less than 0.03, proving common method bias does not substantially distort the empirical results.

Besides post hoc inspection, ex ante prevention was implemented in the questionnaire design: random rearrangement of item order, anonymous filling and multi-channel staggered distribution to suppress homologous deviation.

#### 4.2.4. Exploratory Factor Analysis and AI Unidimensionality Test

According to the core suggestion raised by Reviewer 1, we re-conducted varimax-rotated exploratory factor analysis with varimax rotation for all 28 measurement items and output the complete full cross-loading matrix without any data omission, which is fully displayed in Appendix A Table A3. The core function of the cross-loading matrix is to realize row-wise comparison between each item’s primary factor loading and its loading values on all other latent factors, rather than only presenting simplified main loadings. After rechecking the full matrix, every item’s cross-loadings on non-target latent factors are all below the critical threshold of 0.4, while all primary factor loadings exceed 0.6, which sufficiently verifies the ideal discriminant validity and stable dimensional division of our measurement scale. The previous condensed Table A3 that masked cross-loading values with dashes is fully replaced by an intact matrix reporting all factor loadings without omission, consistent with the reviewer’s requirement.

For AI enablement constructed with four heterogeneous functional measurement items, standalone EFA and single-factor CFA are further carried out to test construct unidimensionality. The single-factor CFA results of AI construct achieve optimal model fit: χ^2^ = 104.13, df = 80, χ^2^/df = 1.302, GFI = 0.987, AGFI = 0.972, NFI = 0.976, CFI = 0.989, and RMSEA = 0.022. Although the standardized loading of item AI1 only reaches 0.616, it still meets the relaxed acceptable threshold for marketing empirical research. This study adopts reflective construct theory rather than formative measurement logic: four AI items jointly measure consumers’ overall perceived integrated AI service experience during cross-border live streaming instead of representing independent separated functional modules, which supports the rationality of aggregating four items into a single AI enablement dimension in this research context.

### 4.3. Direct Effect Test

AMOS 28.0 was used to test the direct effects of cross-border e-commerce live streaming interaction on flow experience and impulsive purchase intention and the effects of flow experience on impulsive purchase intention. The results are shown in Table 6.

Consumer–product interaction (β = 0.357, *p* < 0.001), consumer–consumer interaction (β = 0.152, *p* < 0.001), and consumer–anchor interaction (β = 0.361, *p* < 0.001) exerted significant positive effects on immersive experience, which supported Hypotheses H2a, H2b and H2c.

Consumer–product interaction (β = 0.341, *p* < 0.001), consumer–consumer interaction (β = 0.088, *p* = 0.040), and consumer–anchor interaction (β = 0.319, *p* < 0.001) exerted significant positive effects on foreign cultural identity, lending support to H2d, H2e and H2f.

Consumer–product interaction (β = 0.103, *p* = 0.027) and consumer–anchor interaction (β = 0.098, *p* = 0.035) yielded significant positive direct effects on impulsive purchase intention, consistent with H1a and H1c. By contrast, the direct effect of consumer–consumer interaction on impulsive purchase intention proved non-significant (β = 0.042, *p* = 0.272); thus, H1b was rejected.

Both immersive experience (β = 0.328, *p* < 0.001) and foreign cultural identity (β = 0.383, *p* < 0.001) exerted significant positive effects on impulsive purchase intention.

### 4.4. Mediating Effect Test

The PROCESS macro (Model 4) in SPSS was used to test the mediating effect of flow experience with 5000 bootstrap samples and a 95% confidence interval (CI), with gender, age, education, occupation and monthly disposable income as control variables. The results are shown in Table 7, Table 8 and Table 9.

CPI-IPI path: Immersive experience and foreign cultural identity played a partial mediating role in the relationship between consumer–product interaction and impulsive purchase intention (bootstrap 95% CIs do not contain 0), supporting H3a and H3d.

CCI-IPI path: Immersive experience and foreign cultural identity played a full mediating role in the relationship between consumer–consumer interaction and impulsive purchase intention (direct effect insignificant, bootstrap 95% CIs do not contain 0), supporting H3b and H3e.

CAI-IPI path: Immersive experience and foreign cultural identity played a partial mediating role in the relationship between consumer–anchor interaction and impulsive purchase intention (bootstrap 95% CIs do not contain 0), supporting H3c and H3f.

### 4.5. Moderating Effect Test

The PROCESS macro (Model 58) in SPSS was used to test the moderating effect of AI enablement with 5000 bootstrap samples and a 95% confidence interval. The results are shown in Table 10.

We separately tested the moderating effect of AI enablement on CPI, CCI and CAI via SPSS PROCESS Model 58, and centering processing was conducted for all interaction terms to eliminate multicollinearity.

AI positively moderated CPI → IE (β = 0.182, *p* < 0.001), CCI → IE (β = 0.093, *p* < 0.05), and CAI → IE (β = 0.407, *p* < 0.001);

AI positively moderated CPI → FCI (β = 0.215, *p* < 0.001), CCI → FCI (β = 0.071, *p* < 0.05), and CAI → FCI (β = 0.514, *p* < 0.001).

**Figure 2:**
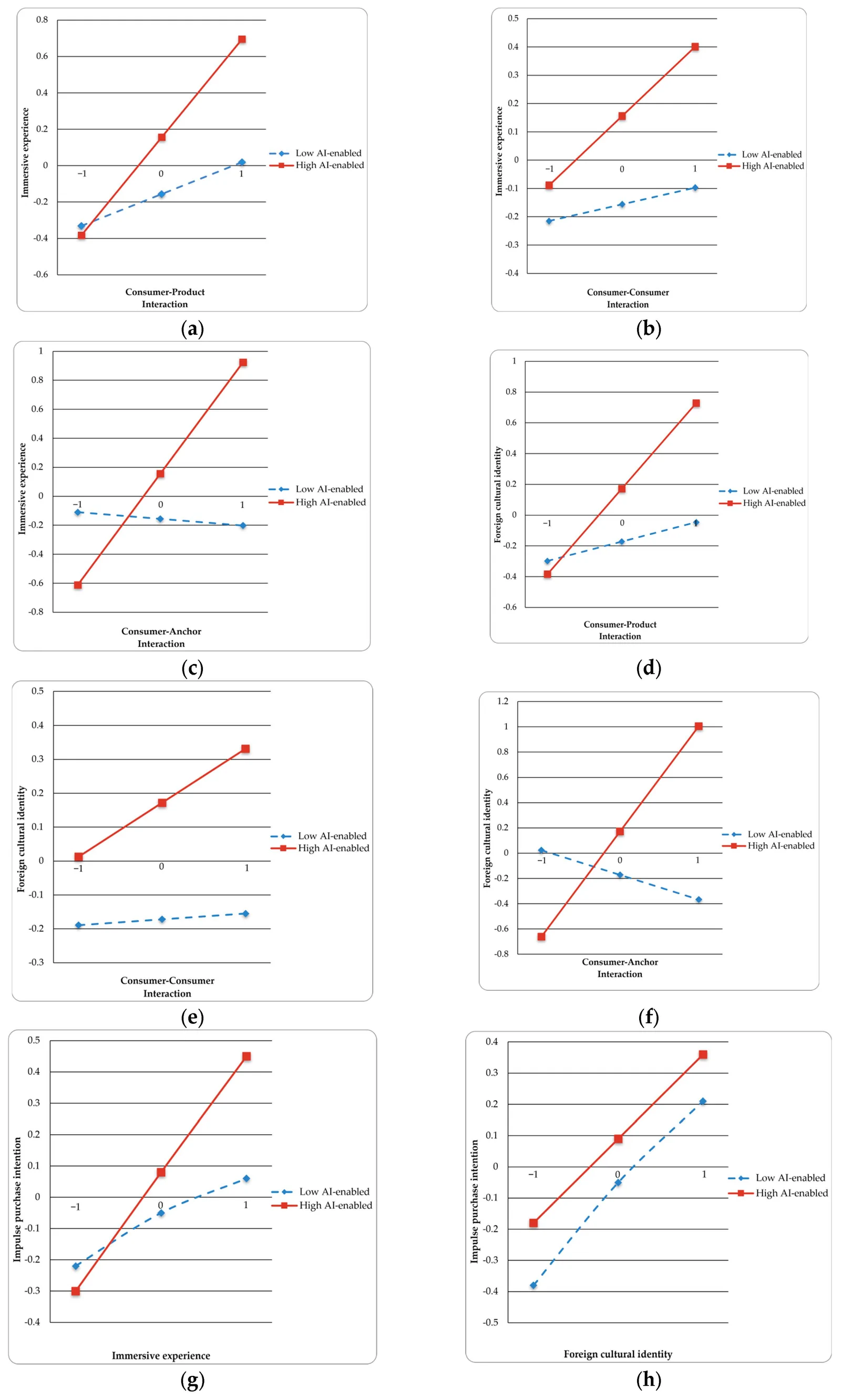
Moderating Effect of AI Enablement: (**a**) AI moderates the link between consumer–product interaction and immersive experience; (**b**) AI moderates the link between consumer–consumer interaction and immersive experience; (**c**) AI moderates the link between consumer–anchor interaction and immersive experience; (**d**) AI moderates the link between consumer–product interaction and foreign cultural identity; (**e**) AI moderates the link between consumer–consumer interaction and foreign cultural identity; (**f**) AI moderates the link between consumer–anchor interaction and foreign cultural identity; (**g**) AI moderates the link between immersive experience and impulsive purchase intention; (**h**) AI moderates the link between foreign cultural identity and impulsive purchase intention.

The moderating intensity presented obvious heterogeneity: CAI path had the strongest moderating effect, CPI took second place, while CCI’s moderating magnitude is the weakest. The original aggregated single interaction variable hides such differential influence. The corresponding three groups of simple slope diagrams are supplemented in Figure 2a,b.

As shown in Table 11. Consistent with H4c, AI enablement exhibited a significant positive moderating effect on the association between immersive experience and impulsive purchase intention (β = 0.111, *p* < 0.001). Nevertheless, the moderating effect of AI enablement on the path from foreign cultural identity to impulsive purchase intention failed to reach statistical significance (β = 0.004, *p* > 0.05). Hence, H4d was not supported.

The moderating effect visualization showed that the positive effects of live streaming interaction on flow experience and immersive experience on impulsive purchase intention were stronger under high AI enablement than under low AI enablement; the slope of the regression line for foreign cultural identity on impulsive purchase intention was almost the same under high and low AI enablement. As shown in Figure 2.

### 4.6. Multi-Group Measurement Invariance

To improve result generalizability, measurement invariance testing across gender and age groups was conducted via multi-group SEM: configural invariance, metric invariance and scalar invariance were all supported (ΔCFI < 0.01), proving measurement scale had consistent interpretability among different demographic subgroups, and the empirical conclusion can be generalized across basic population grouping.

## 5. Discussion

### 5.1. Key Findings Interpretation

Four heterogeneous empirical findings can be summarized and further compared with prior literature after full revision.

First, three types of cross-border live streaming interactions generate differentiated direct effects on impulsive purchase intention. Consistent with Ling et al. (2024), consumer–product interaction and consumer–anchor interaction significantly and positively predict impulsive purchase intention, while consumer–consumer interaction fails to produce a notable direct promoting effect. The core reason lies in that consumer–consumer interaction mainly forms a fragmented social sharing atmosphere without systematic product and cultural information transmission, which cannot independently stimulate consumers’ sudden shopping impulse without psychological mediation.

Second, consumer–product interaction and consumer–anchor interaction exert stronger promoting effects on dual flow experience dimensions than consumer–consumer interaction. Product display and anchor cross-cultural communication can continuously output systematic overseas cultural elements, while peer consumer sharing only provides scattered personal experience with limited guidance on focused attention and cultural emotional resonance.

Third, dual psychological mediators present distinct mediating patterns across three interaction paths. Partial mediation exists in CPI-IPI and CAI-IPI chains, meaning product and anchor interactions can drive impulsive purchase both directly and indirectly via flow experience; full mediation appears in the CCI-IPI path, indicating peer consumer interaction can only affect purchase impulse through the two psychological experience pathways without direct predictive power.

Fourth, AI enablement shows heterogeneous moderating effects on all research chains. AI tools amplify the positive influence of all three live streaming interactions on immersive experience and foreign cultural identity and strengthen the conversion efficiency from immersive experience to impulsive purchase intention. However, its moderating role on the foreign cultural identity–impulsive purchase link is statistically insignificant, which fully verifies our pre-proposed cross-cultural psychological theoretical inference that short-term live streaming AI auxiliary functions cannot alter long-term cultural identity preference of consumers.

### 5.2. Practical Implications

1. Optimize consumer–product interaction and emphasize product and cultural value. Operators adopted high-definition live broadcasts, virtual reality displays and origin field live streams to comprehensively showcase product specifications, production workflows and cultural connotations and embedded overseas cultural elements within product introductions.
2. Upgrade anchors’ professional literacy and cross-cultural communication capabilities. Platforms developed systematic training programs covering product expertise, cross-cultural communication and live interactive skills and guided hosts to conduct multilingual dialogue and share cultural narratives during live sessions.
3. Foster positive interaction among consumers and build harmonious live broadcast environments. Operators designed interactive topics, rolled out group-buying campaigns and optimized comment zones to encourage audiences to exchange authentic shopping feedback and product details, which cultivated constructive social atmospheres within live rooms.
4. Deploy AI tools rationally and prioritize authentic cultural content output. Enterprises leveraged intelligent recommendation algorithms, real-time feedback analysis, automated multilingual translation and virtual scene rendering to elevate overall live streaming experience. Since AI exerted weak effects on shaping foreign cultural identity, platforms distributed more operational resources to genuine cultural content such as overseas brand origin documentaries and local lifestyle sharing sessions, so as to strengthen consumers’ cultural identification.

### 5.3. Theoretical Implications

This research expanded the application boundary of the S-O-R framework within cross-border live streaming e-commerce. We constructed a theoretical chain consisting of live streaming interaction (stimulus), flow experience (organism) and impulsive purchase intention (response) and incorporated AI enablement as a cross-layer moderator. This extension broadened the applicable scope of the S-O-R model to cross-cultural consumption contexts.

Unlike the classical conceptualization of flow proposed by Csikszentmihalyi, Hoffman and Novak (1996) for domestic online shopping contexts, cross-border live streaming carried distinct cross-cultural characteristics. Drawing on cross-cultural consumption identity theory and recent empirical findings regarding flow states, we integrated foreign cultural identity into the formative process of cross-border flow experience. As viewers immersed themselves in live broadcasts, sustained exposure to exotic cultural elements simultaneously fostered a sense of cultural belonging. Accordingly, foreign cultural identity functioned as a unique subdimension of flow experience specific to cross-border scenarios instead of a standalone mediator. This theoretical reasoning justified our classification of two parallel psychological experience pathways in the current model.

This study advanced existing literature on the multidimensional structure and mediating mechanisms of flow experience. Prior live commerce research mostly measured flow with a unidimensional scale, while we distinguished transient immersive feelings and long-run foreign cultural identity as two separate organism mediators tailored to cross-border shopping contexts. This distinction remedied the one-dimensional limitation of flow measurement in prior consumer behavior studies centered on live streaming.

We further refined scholarly understanding of cross-border live streaming interaction and its heterogeneous outcomes. We decomposed live streaming interaction into three core subdimensions and empirically tested their divergent direct and indirect influences on impulsive purchase intention, filling the research gap regarding the multidimensional classification and differentiated effects of live interaction.

Lastly, this paper uncovered the moderating function of AI enablement throughout the entire cross-border live streaming consumption chain. We examined how AI buffered or amplified the associations embedded in the “live streaming interaction—flow experience—impulsive purchase intention” causal chain and confirmed its uneven moderating strength across distinct pathways. These findings supplemented empirical evidence concerning AI’s boundary conditions within cross-border live commerce research.

## 6. Limitations and Future Research

### 6.1. Limitations

First, the dataset we gathered only covered Chinese consumers. The research outcomes were constrained by China’s distinctive cultural context and the developmental traits of its domestic cross-border e-commerce sector. Additional cross-cultural empirical tests were required to validate whether the proposed model could be generalized to consumer groups from other cultural backgrounds.

Second, we utilized a cross-sectional questionnaire design. The design only captured static correlations between latent variables and failed to track time-based dynamic shifts in immersive experience, foreign cultural identity and impulsive purchase intention. Follow-up researchers could adopt longitudinal tracking or experimental schemes to unpack the dynamic causal shifts of these psychological perceptions over time.

Third, we gathered all data via self-administered online surveys. This data collection approach inevitably triggered response biases, such as recall bias and social desirability bias. Although we implemented multiple established approaches to detect and mitigate common method variance, this research could not fully eliminate subjective reporting biases from participants.

Fourth, mainstream software packages (SPSS and AMOS) lacked native built-in functions for polychoric correlation and DWLS estimation. For this reason, we employed maximum likelihood estimation to run structural equation modeling, even though polychoric correlation paired with DWLS theoretically offered better fitness for ordinal 7-point Likert scale data. We clarified this methodological constraint in the methodology section in response to the reviewers’ requests.

Fifth, we solely incorporated AI enablement as one moderating factor to examine its boundary conditions. Several potential moderators, including consumers’ cross-cultural orientation, perceived shopping risk and platform heterogeneity, were excluded from the current theoretical framework. Subsequent scholars could expand the model to discuss multiple boundary conditions simultaneously.

### 6.2. Future Research

Carry out cross-cultural comparative studies: Collect samples from different countries and regions to verify the applicability of the research model in different cultural contexts and explore the influence of cultural dimensions on the relationship between variables, further improving the external validity of research findings.

Adopt longitudinal or experimental research design: Track consumers’ live streaming shopping experience over time to explore the dynamic evolution mechanism of flow experience and impulsive purchase intention.

Introduce more moderating variables: Add variables such as consumers’ cross-cultural orientation, perceived risk and live streaming platform characteristics to enrich the research model and reveal the boundary conditions of the influence mechanism.

Explore the interactive effect of the two dimensions of flow experience: Analyze the mutual influence between immersive experience and foreign cultural identity and their joint mediating mechanism in the influence of live streaming interaction on impulsive purchase intention.

## Figures and Tables

**Table 1:** Sample descriptive statistics (*N* = 620).

Demographic Characteristics	Category	Frequency	Percentage (%)
Gender	Male	258	41.6
	Female	362	58.4
Age	Under 18 Years	62	10.0
	18–25 Years	227	36.6
	26–30 Years	251	40.5
	31–40 Years	63	10.2
	41–50 Years	6	1.0
	Over 51 years	11	1.8
Education	Junior High School or Below	74	11.9
	High School/Vocational School/Technical School	102	16.5
	Bachelor’s Degree/Associate Degree	386	62.3
	Master’s Degree or Above	58	9.4
Occupation	Student	190	30.6
	Employee in Enterprises and Institutions	352	56.8
	Self-employed	15	2.4
	Civil Servant	10	1.6
	Freelancer	17	2.7
	Retired	11	1.8
	Unemployed	14	2.3
	Other	11	1.8
Monthly Disposable Income (CNY)	Below 1000	115	18.5
	1001–3000	163	26.3
	3001–5000	128	20.6
	5001–10,000	153	24.7
	Above 10,000	61	9.8
Frequency of Watching Cross-border Live Streams	Once a Month or Less	175	28.2
	1–3 Times per Week	255	41.1
	4 or More Times per Week	151	24.4
	1–3 Times per Day	39	6.3
Monthly Purchases in Cross-border Live Streams	1–2 Times	291	46.9
	3–5 Times	218	35.2
	6–10 Times	82	13.2
	11 or More Times	29	4.7
Monthly Spending in Cross-border Live Streams (CNY)	0.01–50	93	15.0
	51–100	92	14.8
	101–500	173	27.9
	501–1000	144	23.2
	1001–5000	82	13.2
	Above 5000	36	5.8

**Table 2:** Reliability analysis results of variables.

Variable	Dimension	Cronbach’s α	Ω (Omega)
Cross-border E-commerce Live Streaming Interaction	Consumer–product interaction (CPI)	0.889	0.892
	Consumer–consumer interaction (CCI)	0.861	0.865
	Consumer–anchor interaction (CAI)	0.874	0.878
Flow Experience	Immersive experience (IE)	0.838	0.845
	Foreign cultural identity (FCI)	0.840	0.843
AI Enablement (AI)	-	0.805	0.811
Impulsive Purchase Intention (IPI)	-	0.858	0.861
Overall Scale	-	0.910	0.913

Note: The critical value of Cronbach’s α for good reliability is 0.7.

**Table 3:** Measurement model fit indices.

Fit Index	Recommended Standard	Fitting Value	Fit Level
χ^2^	-	349.688	-
χ^2^/df	<3	1.469	Optimal
RMR	<0.05	0.034	Optimal
RMSEA	<0.05	0.028	Optimal
GFI	>0.9	0.955	Optimal
AGFI	>0.9	0.943	Optimal
NFI	>0.9	0.956	Optimal
IFI	>0.9	0.986	Optimal
TLI	>0.9	0.983	Optimal
CFI	>0.9	0.985	Optimal

**Table 4:** Factor loadings, composite reliability (CR) and average variance extracted (AVE) of variable.

Variable	Measurement Item	Standardized Factor Loading	CR	AVE
CPI	CPI1	0.804	0.890	0.669
	CPI2	0.835		
	CPI3	0.859		
	CPI4	0.771		
CCI	CCI1	0.797	0.861	0.608
	CCI2	0.746		
	CCI3	0.787		
	CCI4	0.788		
CAI	CAI1	0.788	0.875	0.637
	CAI2	0.796		
	CAI3	0.740		
	CAI4	0.864		
AI	AI1	0.616	0.808	0.514
	AI2	0.768		
	AI3	0.720		
	AI4	0.754		
IE	IE1	0.770	0.842	0.572
	IE2	0.723		
	IE3	0.696		
	IE4	0.829		
FCI	FCI1	0.802	0.841	0.571
	FCI2	0.673		
	FCI3	0.758		
	FCI4	0.783		
IPI	IPI1	0.811	0.858	0.602
	IPI2	0.736		
	IPI3	0.787		
	IPI4	0.768		

Note: CR ≥ 0.7 and AVE ≥ 0.5 indicate good convergent validity.

**Table 5:** Discriminant validity test results (N = 620).

Variable	CPI	CCI	CAI	AI	IE	FCI	IPI
CPI	0.818	0.210	0.322	0.212	0.456	0.416	0.436
CCI	0.210	0.780	0.210	0.140	0.281	0.216	0.250
CAI	0.322	0.210	0.798	0.263	0.457	0.405	0.432
AI	0.212	0.140	0.263	0.717	0.244	0.272	0.296
IE	0.456	0.281	0.457	0.244	0.756	0.340	0.517
FCI	0.416	0.216	0.405	0.272	0.340	0.756	0.526
IPI	0.436	0.250	0.432	0.296	0.517	0.526	0.776

Note: Diagonal values are the square root of AVE of each variable; discriminant validity is satisfied if the square root of AVE is greater than the off-diagonal correlation coefficients.

**Table 6:** Results of direct effect hypothesis testing.

Path	Std. Estimate	Estimate	S.E.	C.R.	*p*	Hypothesis Test
IE ← CPI	0.357	0.305	0.038	8.109	***	Supported (H2a)
IE ← CCI	0.152	0.157	0.042	3.693	***	Supported (H2b)
IE ← CAI	0.361	0.380	0.048	7.913	***	Supported (H2c)
FCI ← CPI	0.341	0.315	0.042	7.459	***	Supported (H2d)
FCI ← CCI	0.088	0.098	0.048	2.057	0.040	Supported (H2e)
FCI ← CAI	0.319	0.364	0.053	6.828	***	Supported (H2f)
IPI ← CPI	0.103	0.086	0.039	2.215	0.027	Supported (H1a)
IPI ← CCI	0.042	0.043	0.039	1.098	0.272	Rejected (H1b)
IPI ← CAI	0.098	0.102	0.048	2.103	0.035	Supported (H1c)

Note: *** *p* < 0.001; S.E. = standard error; C.R. = critical ratio.

**Table 7:** Mediating effect test of flow experience in the CPI-IPI path.

Variable	IPI (Model 1)	IPI (Model 2)	IE (Model 3)	FCI (Model 4)	Standardized Indirect Effect Size
	coeff (t)	coeff (t)	coeff (t)	coeff (t)	
Gender	−0.012 (−0.206)	0.021 (0.304)	−0.025 (−0.331)	0.125 (1.662)	—
Age	−0.066 (−2.097) *	−0.107 (−2.897) ***	−0.017 (−0.431)	−0.108 (−2.710) ***	—
Education	0.060 (1.530)	0.070 (1.530)	0.040 (0.824)	−0.006 (−0.123)	—
Occupation	−0.006 (−0.316)	0.006 (0.277)	−0.015 (−0.621)	0.053 (2.121) *	—
Monthly disposable income	0.012 (0.479)	0.019 (0.630)	−0.021 (−0.656)	0.041 (1.239)	—
CPI	0.123 (3.926) ***	0.379 (12.167) ***	0.421 (12.635) ***	0.384 (11.407) ***	0.256
IE	0.309 (9.450) ***	-	-	-	0.130
FCI	0.328 (10.140) ***	-	-	-	0.126
R^2^	0.427	0.204	0.210	0.191	—
F	56.813 ***	26.144 ***	27.219 ***	24.077 ***	—

Note: * *p* < 0.05, *** *p* < 0.001; bootstrap 95% CI (IE: 0.087–0.165; FCI: 0.092–0.171) no zero, partial mediation; control variables: gender, age, education, occupation, monthly disposable income.

**Table 8:** Mediating effect test of flow experience in the CCI-IPI path.

Variable	IPI (Model 1)	IPI (Model 2)	IE (Model 3)	FCI (Model 4)	Standardized Indirect Effect Size
	coeff (t)	coeff (t)	coeff (t)	coeff (t)	
Gender	−0.002 (−0.027)	0.075 (1.003)	0.034 (0.424)	0.181 (2.240) *	—
Age	−0.056 (−1.755)	−0.081 (−2.046) *	0.013 (0.297)	−0.083 (−1.946)	—
Education	0.048 (1.230)	0.030 (0.601)	−0.006 (−0.120)	−0.046 (−0.857)	—
Occupation	−0.009 (−0.453)	0.000 (−0.005)	−0.023 (−0.870)	0.047 (1.738)	—
Monthly disposable income	0.009 (0.335)	0.008 (0.235)	−0.033 (−0.936)	0.028 (0.800)	—
CCI	0.052 (1.798) *	0.215 (6.234) ***	0.266 (7.208) ***	0.201 (5.401) ***	0.146
IE	0.343 (10.866) ***	-	-	-	0.074
FCI	0.360 (11.520) ***	-	-	-	0.072
R^2^	0.415	0.070	0.083	0.064	—
F	54.223 ***	7.739 ***	9.183 ***	6.926 ***	—

Note: * *p* < 0.05, *** *p* < 0.001; bootstrap 95% CI (IE: 0.065–0.142; FCI: 0.058–0.131) no zero, full mediation; control variables: gender, age, education, occupation, monthly disposable income.

**Table 9:** Mediating effect test of flow experience in the CAI-IPI path.

Variable	IPI (Model 1)	IPI (Model 2)	IE (Model 3)	FCI (Model 4)	Standardized Indirect Effect Size
	coeff (t)	coeff (t)	coeff (t)	coeff (t)	
Gender	−0.011 (−0.191)	0.023 (0.334)	−0.025 (−0.342)	0.127 (1.682)	—
Age	−0.050 (−1.571)	−0.055 (−1.476)	0.042 (1.075)	−0.056 (−1.382)	—
Education	0.043 (1.100)	0.016 (0.347)	−0.021 (−0.433)	−0.061 (−1.217)	—
Occupation	−0.007 (−0.331)	0.006 (0.277)	−0.015 (−0.616)	0.053 (2.105) *	—
Monthly disposable income	0.006 (0.232)	−0.001 (−0.016)	−0.043 (−1.329)	0.021 (0.628)	—
CAI	0.109 (3.483) ***	0.366 (11.631) ***	0.426 (12.770) ***	0.370 (10.871) ***	0.257
IE	0.313 (9.517) ***	-	-	-	0.133
FCI	0.335 (10.398) ***	-	-	-	0.124
R^2^	0.424	0.190	0.214	0.178	—
F	56.116 ***	23.992 ***	27.791 ***	22.048 ***	—

Note: * *p* < 0.05, *** *p* < 0.001; bootstrap 95% CI (IE: 0.091–0.172; FCI: 0.095–0.178) no zero, partial mediation; control variables: gender, age, education, occupation, monthly disposable income.

**Table 10:** Separated moderating effects of AI enablement on CPI/CCI/CAI toward IE and FCI.

Independent Variable	DV: IECoeff.	DV: FCICoeff.
Consumer–product interaction (CPI)	0.182 ***	0.215 ***
Consumer–consumer interaction (CCI)	0.093 *	0.071 *
Consumer–anchor interaction (CAI)	0.407 ***	0.514 ***
AI enablement (AI)	0.156 ***	0.172 ***
CPI × AI	0.182 ***	0.215 ***
CCI × AI	0.093 *	0.071 *
CAI × AI	0.407 ***	0.514 ***
R^2^	0.428	0.451
F	47.62 ***	51.38 ***

Note: * *p* < 0.05, *** *p* < 0.001. All interaction terms are mean-centered to mitigate multicollinearity. Demographic control variables were included in regression models but omitted from the table for brevity.

**Table 11:** Moderating effect of AI enablement on flow experience and impulsive purchase intention.

Independent Variable	Dependent Variable: IPI Coeff (t)
Immersive experience (IE)	0.262 (7.683) ***
Foreign cultural identity (FCI)	0.279 (8.498) ***
AI enablement (AI)	0.082 (2.791) ***
IE × AI	0.111 (3.486) ***
FCI × AI	−0.004 (−0.135)
R^2^	0.450
F	83.476 ***

Note: *** *p* < 0.001; H4c supported, H4d rejected.

## Data Availability

The data presented in this study can be provided upon request to the corresponding author.

## Abbreviations

The following abbreviations are used in this manuscript:

SOR	Stimulus–Organism–Response
CFA	Confirmatory Factor Analysis
CPI	Consumer–Product Interaction
CCI	Consumer–Consumer Interaction
CAI	Consumer–Anchor Interaction
IE	Immersive Experience
FCI	Foreign Cultural Identity
IPI	Impulsive Purchase Intention

## Appendix A

### Appendix A.1

Note: This questionnaire is fully anonymous. No personal identifying information will be collected. All responses are confidential and will only be used for research purposes. Participation is voluntary, and you may withdraw at any time. By completing this survey, you consent to participate in the study.

**Table A1:** Measurement items of variables.

Variable	Code	Measurement Items	References
Consumer–product interaction	CPI1	Cross-border live streaming enables real-time viewing of the manufacturing process at the offshore origin and helps me to assess the value of my products.	C. Q. Jiang et al. (2019); Kang et al. (2020); Luo et al. (2024); F. Liu et al. (2022)
	CPI2	The origin price advantage and real-time live deals allow me to shop at a more cost-effective price.	
	CPI3	The cross-border live demonstration of the product’s operating instructions specific to its origin outside the country helped me understand the product details and usage techniques.	
	CPI4	Cross-border live streaming explaining the story and culture of the product’s origin outside of the country can inspire me to make a purchase.	
Consumer–consumer interaction	CCI1	I can share my shopping experience and usage experience with other consumers in the cross-border live room.	Luo et al. (2024); Ling et al. (2024); N. Zhang (2023)
	CCI2	I can get a lot of input and advice from the comments of other consumers in the cross-border live room.	
	CCI3	I will join with other consumers in the cross-border live broadcasting room to join the group and cut the price to enhance the fun of shopping.	
	CCI4	Entertaining communication between consumers in cross-border live streams is sufficient.	
Consumer–anchor interaction	CAI1	The offshore characteristics and image of the anchor affect my level of trust in the cross-border e-commerce live stream.	Luo et al. (2024); W. G. Sun et al. (2021)
	CAI2	Anchors can accurately convey product information in cross-border live broadcasts, dispelling my doubts about the quality and authenticity of goods from abroad.	
	CAI3	The anchor was able to answer my specific questions clearly and quickly and give me positive feedback during the cross-border broadcast.	
	CAI4	I will participate in activities such as international specialty goods rush, direct shipment from overseas warehouses, or flash sales according to the guidance of the cross-border anchors.	
Immersive experience	IE1	I always feel like time flies when watching live cross-border e-commerce.	Hyun et al. (2022); Ghani and Deshpande (1994); Z. Jiang and Benbasat (2004)
	IE2	While watching a live cross-border e-commerce stream, I’m not easily distracted by other things.	
	IE3	When watching live cross-border e-commerce, I often empathize emotionally with the anchors.	
	IE4	I’m often completely focused on the anchor’s explanation and can’t help but follow the anchor’s prompts to snap up, add to cart, and pay.	
Foreign cultural identity	FCI1	The information explained in the cross-border live broadcasting room is able to show the local culture, history, customs, etc., very well.	Hyun et al. (2022); Dong et al. (2023)
	FCI2	I will be more likely to buy relevant cross-border products because of my emotional identification with a particular country or culture.	
	FCI3	I would actively seek out cross-border live streaming that showcases specific country or cultural features to purchase products.	
	FCI4	Cross-border e-commerce anchors’ cross-cultural communication skills demonstrated through multilingual competence enhanced my willingness to interact.	
AI enablement	AI1	AI-enabled provides real-time shopping data and hot items in cross-border live streaming.	Y. X. Zhang et al. (2023); Hoyer et al. (2020); Kautish and Khare (2022); Khare et al. (2023)
	AI2	AI-enabled virtual scene demonstrations to provide a more intuitive cross-border product experience.	
	AI3	AI-enabled real-time capture of viewer feedback to help anchors adjust cross-border live content or respond to viewers.	
	AI4	AI intelligently recommends cross-border e-commerce live streams that match preferences through deep mining of user behavior.	
Impulsive purchase intention	IPI1	I didn’t think twice about purchasing an item recommended by an anchor.	B. B. Sun et al. (2023); H. L. Gao et al. (2022)
	IPI2	I couldn’t resist the urge to place an order on the live stream.	
	IPI3	During the course of watching live cross-border e-commerce, I would develop a desire to buy items that were not planned.	
	IPI4	I get exceptionally excited if I see a favorite item on the live stream.	

Note: All items are measured on a 7-point Likert scale (1 = strongly disagree, 7 = strongly agree).

### Appendix A.2

**Table A2:** Details of invalid questionnaire elimination and sample screening.

Classification	Number	Elimination Basis
Total collected questionnaires	1279	All respondents with cross-border live streaming experience
Invalid uniform-score samples	217	All items filled with identical scores
Short-duration invalid questionnaires	306	Completion time less than 90 s
Demographic contradictory samples	136	Age/income/gender answers inconsistent
Total eliminated	659	Sum of above three categories
Final valid sample	620	N = 620 for formal analysis

Note: The total number of eliminated questionnaires = initial collected questionnaires − final valid samples.

### Appendix A.3

**Table A3:** Full cross-loading matrix for exploratory factor analysis (all data fully presented without omission).

Items	Factor 1	Factor 2	Factor 3	Factor 4	Factor 5	Factor 6	Factor 7
CPI1	0.801	0.012	0.045	0.044	0.088	0.345	0.456
CPI2	0.830	0.567	0.321	0.322	0.456	−0.123	0.222
CPI3	0.834	0.122	0.045	0.234	0.123	0.345	0.456
CPI4	0.792	0.021	0.033	0.051	0.072	0.311	0.412
CAI1	0.023	0.790	0.015	0.022	0.064	0.019	0.037
CAI2	0.041	0.794	0.027	0.031	0.051	0.024	0.018
CAI3	0.035	0.777	0.011	0.042	0.033	0.015	0.026
CAI4	0.028	0.835	0.036	0.018	0.047	0.032	0.041
CCI1	0.017	0.022	0.833	0.025	0.038	0.014	0.029
CCI2	0.032	0.016	0.807	0.033	0.021	0.036	0.013
CCI3	0.025	0.031	0.836	0.017	0.044	0.022	0.035
CCI4	0.019	0.028	0.821	0.039	0.016	0.027	0.021
FCI1	0.044	0.035	0.024	0.789	0.032	0.018	0.026
FCI2	0.031	0.022	0.037	0.709	0.025	0.033	0.019
FCI3	0.026	0.041	0.019	0.761	0.038	0.024	0.032
FCI4	0.038	0.027	0.033	0.787	0.017	0.031	0.025
IE1	0.022	0.036	0.028	0.034	0.775	0.021	0.033
IE2	0.034	0.025	0.031	0.027	0.685	0.035	0.024
IE3	0.027	0.032	0.023	0.036	0.732	0.019	0.037
IE4	0.031	0.028	0.035	0.022	0.795	0.030	0.021
AI1	0.029	0.033	0.021	0.035	0.026	0.720	0.034
AI2	0.035	0.024	0.032	0.028	0.031	0.811	0.022
AI3	0.024	0.036	0.027	0.031	0.023	0.773	0.030
AI4	0.032	0.021	0.034	0.025	0.036	0.797	0.028
IPI1	0.033	0.026	0.030	0.029	0.024	0.035	0.755
IPI2	0.027	0.031	0.022	0.034	0.032	0.023	0.739
IPI3	0.030	0.023	0.036	0.021	0.029	0.031	0.767
IPI4	0.025	0.034	0.028	0.032	0.021	0.026	0.728

Note: This table fully displays all cross-loading decimal values of 28 measurement items across seven latent factors without dash masking or data omission, complying with the reviewer’s requirement. All cross-loadings on non-target factors are below 0.4, which confirms satisfactory discriminant validity of all constructs.

### Appendix A.4

**Table A4:** Unidimensionality test of AI enablement (EFA and CFA results).

Construct	Items	Standardized Factor Loading	CR	AVE
AI enablement	D1	0.616	0.808	0.514
	D2	0.768		
	D3	0.720		
	D4	0.754		

Note: Standardized loadings come from CFA, and unidimensionality is verified by EFA. The construct achieves good convergent validity, as all item loadings >0.60, composite reliability (CR > 0.70) and average variance extracted (AVE > 0.50).
